# Redox Control of Multidrug Resistance and Its Possible Modulation by Antioxidants

**DOI:** 10.1155/2016/4251912

**Published:** 2016-01-05

**Authors:** Aysegul Cort, Tomris Ozben, Luciano Saso, Chiara De Luca, Liudmila Korkina

**Affiliations:** ^1^Department of Nutrition and Dietetics, Faculty of Health Sciences, Sanko University, İncili Pınar, Gazi Muhtar Paşa Bulvarı, Sehitkamil, 27090 Gaziantep, Turkey; ^2^Department of Biochemistry, Akdeniz University Medical Faculty, Campus, Dumlupınar Street, 07070 Antalya, Turkey; ^3^Department of Physiology and Pharmacology “Vittorio Erspamer”, La Sapienza University of Rome, Piazzale Aldo Moro 5, 00185 Rome, Italy; ^4^Evidence-Based Well-Being (EB-WB) Ltd., 31 Alt-Stralau, 10245 Berlin, Germany; ^5^Centre of Innovative Biotechnological Investigations Nanolab, 197 Vernadskogo Prospekt, Moscow 119571, Russia

## Abstract

Clinical efficacy of anticancer chemotherapies is dramatically hampered by multidrug resistance (MDR) dependent on inherited traits, acquired defence against toxins, and adaptive mechanisms mounting in tumours. There is overwhelming evidence that molecular events leading to MDR are regulated by redox mechanisms. For example, chemotherapeutics which overrun the first obstacle of redox-regulated cellular uptake channels (MDR1, MDR2, and MDR3) induce a concerted action of phase I/II metabolic enzymes with a temporal redox-regulated axis. This results in rapid metabolic transformation and elimination of a toxin. This metabolic axis is tightly interconnected with the inducible Nrf2-linked pathway, a key switch-on mechanism for upregulation of endogenous antioxidant enzymes and detoxifying systems. As a result, chemotherapeutics and cytotoxic by-products of their metabolism (ROS, hydroperoxides, and aldehydes) are inactivated and MDR occurs. On the other hand, tumour cells are capable of mounting an adaptive antioxidant response against ROS produced by chemotherapeutics and host immune cells. The multiple redox-dependent mechanisms involved in MDR prompted suggesting redox-active drugs (antioxidants and prooxidants) or inhibitors of inducible antioxidant defence as a novel approach to diminish MDR. Pitfalls and progress in this direction are discussed.

## 1. Introduction

It is common knowledge that multiple drug resistance (MDR) has crucial negative impact on the clinical outcomes of conventional cytotoxic anticancer therapies and of those based on specific drugs targeting molecular pathways implicated in cancer cell functions and survival strategies. Since the discovery of the first ATP-binding cassette (ABC) transporter P-glycoprotein (P-gp), ABC drug transporters have become targets for improving anticancer chemotherapy. Up to now, more than 49 different ABC transporters have been found and cloned [1]. A majority of MDR modulators or reversals are themselves substrates of the transporters that compete with anticancer agents for the efflux from tumour cells [2]. Frustrating the great expectations raised, ABC transporter/modulators/reversals proved to have insufficient clinical efficacy and very high toxicity. Novel “biological” approaches have been recently developed in laboratory to modulate ABC transporter-mediated MDR, including a monoclonal antibody that binds specifically to P-gp, thus suppressing drug transport, small interfering RNA technology to decrease the expression of ABCB1, antisense oligonucleotides, and agents attenuating P-gp transcription [3]. Though very promising, these “biologicals” are still lacking clinical proof-of-concept data.

In any case, the evident and numerous adverse effects of MDR modulators stimulated additional studies on physiological role(s) of MDR in the human organism. It has been reported that MDR relies not exclusively on transporting systems for drug uptake and efflux, but also on intracellular drug metabolism and DNA damage [4]. Transporting and metabolic systems defining MDR are expressed in the majority of normal cells, are essential for nutrients uptake and metabolites efflux, and play a vital role in protecting cells against xenobiotics. Hence, harsh inhibition of a functionally essential mechanism results in general intoxication.

To gain protection against foreign invasions and maintain homeostasis, the human organism employs several types of physical, chemical, and biological defence systems. For example, skin and other lining epithelia mechanically prevent invasion of relatively large organic and inorganic particles. The immune system has been evolved to fight cellular invaders and high-molecular-weight compounds of biological origin.

The chemical defence system, consisting of biosensoring, transmitting, and responsive elements, has been evolved, starting from primitive eukaryotes and lower plants [5], to protect multicellular organisms against environmental chemical insults (xenobiotics) and to maintain homeostasis of endogenous low-molecular-weight metabolites (endobiotics) [6]. Being exposed to xenobiotic (drug) stress, an organism is challenged to rapid and appropriate adaptation by activating constitutive and expressing inducible systems, thus attenuating negative biological consequences. For this purpose, an array of gene families and molecular pathways have been developed during evolution to prevent cellular access, to detoxify and eliminate toxins, and to repair chemical damage. The active efflux proteins, for example, P-glycoproteins (P-gp) [7], multidrug resistance (MDR) proteins [8], and multixenobiotic resistance (MXR) proteins [9], directly eliminate slightly lipophilic organic xenobiotics from cells serving as the first line of chemical defence. Escaping the first-line guardians, once in the cytoplasm, toxic nucleophilic compounds undergo biotransformation by the oxidative phase I enzymes (cytochrome P450 (CYP), flavoprotein monooxygenase, hemeoxygenase, amine oxidases, xanthine oxidase, and others) to become electrophilic. The electrophile is subjected to reductive or conjugative modification by phase II enzymes (glutathione-S-transferases (GSTs), UDP-glucuronosyltransferases (UGTs), catechol-*O*-methyl transferases (COMT), N-acetyl transferases (NATs), and many others).

Reactive oxygen species (ROS) generated as by-products of phase I reactions are rapidly reduced to nontoxic “physiological” levels by antioxidant enzymes (superoxide dismutases (SODs), catalase (CAT), glutathione peroxidases (GPx), peroxiredoxins (PRx), and nonenzymatic antioxidants, such as reduced glutathione (GSH), uric acid, ascorbic acid, and ceruloplasmin, among others). All these constitutive protective systems are sufficient to cope with low levels of xenobiotics or endobiotics. The inducible chemical defence relies on the array of stress responsive genes. In this case, chemical stressors like anticancer chemotherapeutics should first be recognised by specific sensors which, in turn, transmit alarm signals to activate or express* de novo* transporting, biotransforming, and detoxifying enzymes.

The primary member of mammalian proteins-sensors of organic chemicals is the aryl hydrocarbon receptor (AhR), activated by planar aromatic hydrocarbons of natural or synthetic origin [10–12]. A second group of chemical sensors comprises nuclear receptors, such as pregnane X, constitutive androstane, peroxisome proliferators-activated, liver-X, and farnesoid-X receptors, recognising a wide variety of xeno- and endobiotics [12–14]. Nuclear factor erythroid-derived 2-related factors (Nrf1 and Nrf2) and related cap'n'collar- (CNC-) basic leucine zipper proteins belong to another family of sensors activated by oxidants and electrophiles [15, 16]. Activation of such recognition elements after ligand binding may result in alterations of ion channel conductivity, kinase machinery, and cytoplasmic and nuclear transcription factors, inducing cell response (signal transduction).

Signal transduction is often mediated by redox substances (superoxide anion radical, hydrogen peroxide, lipid peroxides, aldehydes, and others) [17–21]. At moderate concentrations, they are signals to start gene transcription via activation of transcription factors (nuclear factor *κ*B (NF-*κ*B), activator protein 1 (AP-1), and antioxidant response element- (ARE-) binding proteins) or initiating the protein kinase cascade [5, 16, 19]. The latter pathway leads to the interaction with specific ARE of DNA motifs on promoters of antioxidant defence enzymes such as GST, Mn-superoxide dismutase (MnSOD), and glutamyl-cysteine ligase, among others [15].

**Figure 1:**
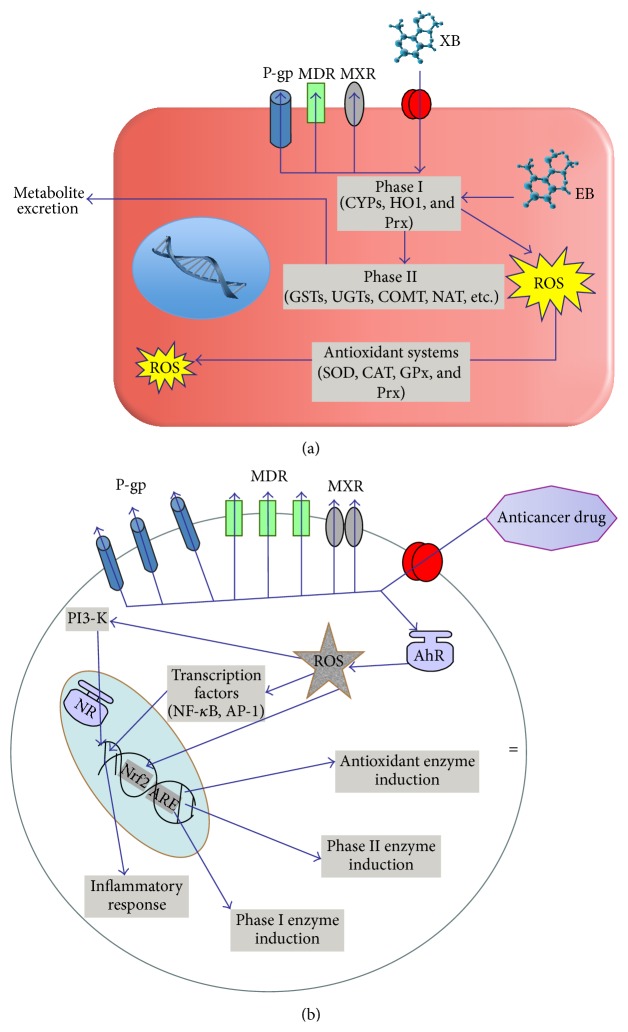
Inherited and acquired multiple drug resistance. (a) In the inherited multiple drug resistance (MDR), chronic exposure of normal cells to low levels of unknown xenobiotics (XB) or/and endobiotics (EB) takes place. It causes upregulation of ATP-binding cassette transporters such as P-glycoprotein (P-gp), MDR proteins (MDRs), and multiple xenobiotic resistance (MXR) without induction by anticancer drugs. Single nucleotide polymorphisms of phase I and II metabolic enzymes and efflux transporters often accompany inherited MDR and they could also be a causative reason for the resistance. Reactive oxygen species-mediated modulation of xenobiotics/drug metabolism is similar to that in the acquired drug resistance. This cellular pattern seems to be associated with high risk of tumour transformation. ROS: reactive oxygen species; MDR: multiple drug resistance transporters; MXR: multiple xenobiotic resistance transporters; P-gp: P-glycoprotein; CYP: cytochrome P450; HO1: hemeoxygenase-1; SOD: superoxide dismutase; CAT: catalase; GPx: glutathione peroxidase; PI3K: phosphatidylinositol-3 kinase; AhR: aromatic hydrocarbon receptor; NF-*κ*B: nuclear factor kappa B; AP-1: activator protein 1; NR: nuclear receptor; Nrf2: nuclear factor erythroid-derived 2-related factor 2; ARE: antioxidant responsive elements. (b) In the acquired MDR, chemotherapeutics induce redox-dependent MDR expression and activity in tumour cells. Chemotherapeutics activate also aromatic hydrocarbon receptor- (AhR-) driven and ROS-regulated expression of transcriptional factors (nuclear factor kappa B (NF-*κ*B) and activator protein 1 (AP-1)) which initiate inflammatory response. Reactive oxygen species (ROS) mediate activation of phosphoinositol-3 kinase upstream of inflammatory cytokine transcription and synthesis. ROS and AhR-associated stimulation of Nrf2 followed by antioxidant responsive element of DNA motif causes upregulation of protective, antioxidant, and detoxifying systems, such as antioxidant phase I and II enzymes.

Inherited or acquired alterations at any key point of the chemical defence system might lead to chronic intoxication and numerous human pathologies (chronic inflammation, degeneration, carcinogenesis, multiple chemical sensitivity syndrome, etc.) in the inherited or acquired MDR, respectively (Figure 1). Usually, the course of anticancer chemotherapy induces the overexpression of drug transporters MDR/MXR/P-gp [3, 4], activation of sensoring receptors, electrophile/oxidant sensors, transcription factors, and overexpression/activation of detoxifying/antioxidant enzymes [1]. Collectively, it causes rapid metabolism and elimination of both the anticancer drugs and cytotoxic by-products targeting tumour cells. Since the chemical defence system is ubiquitous for all human organs and tissues and central to organism functions, the attempts for its pharmacological suppression in order to diminish MDR potentially bear the risk of a multitude of undesired side effects. Upon the pharmacological interaction with components of universal chemical defence system, the “good guy” evolved on purpose to protect multicellular organisms from low-molecular-weight chemicals could become a “bad guy” blocking desired therapeutical effects of anticancer drugs (MDR).

**Figure 2:**
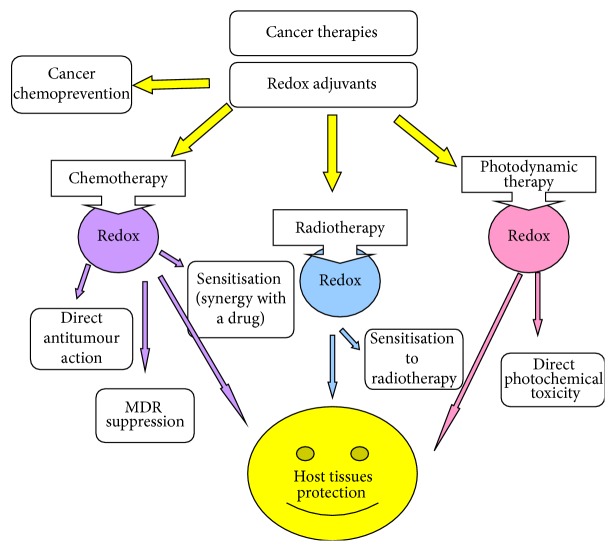
Redox-active substances and cancer. A variety of redox-active substances (direct or indirect antioxidants) are known to exhibit cancer chemopreventive properties. In the pharmacological anticancer protocols, redox-active agents could be used as direct anticancer chemotherapeutics or synergies with cytotoxic effects of conventional anticancer drugs. Here, we discuss the feasibility of such substances in suppression/reversal of acquired MDR. The redox agents are often used for the protection of normal tissues/organs against toxic effects of chemotherapy and radiotherapy.

On the grounds of our current knowledge, redox regulation of multiple molecular pathways essential for human chemical defence system can be implicated differentially in normal host and in tumour cells. Owing to the fact that redox balance in tumour cells is greatly altered as compared to that of normal host cells [22, 23], selective redox inhibitors targeting tumour-associated chemical defence as a cause of MDR should be developed. Regarding potential health effects of redox modulators on tumours, they are mainly attributed to cancer chemoprevention, direct anticancer action (for comprehensive review, see [23]), cancer sensitisation to conventional chemotherapeutics, preferentially through MDR suppression/reversal, cancer sensitisation to radio- and photodynamic therapies, and protection of normal host organs/tissues against damage by chemo- and radiotherapy (Figure 2).

This review will discuss existing and perspective possibilities of differential targeted modulation of redox-dependent components/pathways of intrinsic and induced chemical defence as an emerging strategy for combinatory anticancer therapies to overcome MDR. Molecular pathways-targets for MDR attenuation or even reversal by redox-active substances will be described in detail.

## 2. Intrinsic Multidrug Resistance (MDR): Is It Possible to Overcome It by Redox Modulation?

### 2.1. Inherited Overexpression of Drug Transporters Accelerates Drug Efflux from Target Cells

Some individuals possess the so-called intrinsic MDR having never been exposed to chemotherapy. Genetical predisposition to resist xenobiotic stress could, in principle, be explained in terms of single nucleotide polymorphisms (SNPs) of complex MDR system components, starting from drug transporters, censoring receptors, and xenobiotic/drug metabolising enzymes (see several examples below). On the other hand, a leading hypothesis indicates intrinsic MDR as a result of chronic (“silent”) exposure to low-level xenobiotic stressors or endogenous disturbances of lipid, glucose, and/or hormone metabolism. Therefore, the molecular pathways of its regulations are similar to those characteristic for acquired/chemotherapy-induced MDR. In this line, the development of intrinsic MDR correlates with increased risk of carcinogenesis, and the process is under network-like redox control (for comprehensive review, see [1]). It appears that the classical paradigm of cancer chemoprevention with redox-active nontoxic substances could be interpreted in terms of intrinsic MDR prevention. Furthermore, intrinsic MDR is a hallmark of stem cells, both normal and tumour, because high resistance to any toxin would guarantee survival and maintenance of stem cell populations.

### 2.2. Gene Polymorphisms Influencing Drug Metabolising Enzymes May Result in Ultrafast Drug Elimination or Extremely Slow Formation of Cytotoxic Redox By-Products

The cytochrome P450 (CYP) system is a superfamily of isozymes, located in the smooth endoplasmic reticulum, mainly in the liver, but also in extrahepatic tissues (e.g., intestinal mucosa, lung, kidney, brain, lymphocytes, placenta, and skin), involved in the biotransformation of numerous lipophilic xenobiotics into more hydrophilic, less toxic, and more easily excreted metabolites [11, 24, 25]. The major CYP enzymes involved in human drug metabolism belong to families 1, 2, and 3, the specific drug metabolising isoforms being* Cyp1A2*,* Cyp2C9*,* Cyp2C19*,* Cyp2D6*, and* Cyp3A4/3A5*.

Each CYP isoform is a product of specific gene. For some isoforms, the existence of genetic polymorphisms has been demonstrated. The allelic variants may be due to the deletion of the entire gene, SNPs, deletion or insertion of fragments of DNA within the gene, or multiplication of gene copies, leading to absent, deficient, or enhanced enzyme activity. Thus, the population can be classified into Extensive Metabolisers (EM, individuals with normal capacity), Poor Metabolisers (PM, individuals with reduced/null metabolic activity), and Ultrarapid Metabolisers (UM, individuals with a higher-than-normal metabolic activity). It seems that opposite populations of PM and UM could be at risk of constitutive MDR, because UM would rapidly metabolise/excrete parent molecules of anticancer drugs, while PM would not produce ROS as by-products of anticancer drug metabolism. These by-products possess strong cytotoxicity against cancer cells. Hence, the routine clinical diagnostics based on determination of CYP SNPs produce a reliable prediction of individual chemosensitivity/chemoresistance/MDR to anticancer therapies. Several polymorphisms have been connected with the inducibility or enzymatic activity of the abovementioned drug metabolising CYP isoforms [6].

### 2.3. Redox-Active Inhibitors of Drug Transporters and Receptors Associated with Drug Detoxifying Enzymes: Hopes and Reality

Notwithstanding the growing interest and great hopes for natural nontoxic redox agents to prevent/inhibit/reverse MDR ([26]; in this review), drug development remains rather complicated due to low bioavailability, defined by restricted absorption through intestine, lining epithelia, and skin [24, 25], rapid metabolism, and excretion. Absorbed MDR inhibitors become themselves targets for the classical pathways of xenobiotic detoxification/drug metabolism [27–29]. In phase I, they are predominantly metabolised by microsomal CYPs. Then, phase II glucuronidation by UGT [30], sulfation by phenol and catecholamine specific sulfotransferases (SULT1A1 and SULT1A3) [11], methylation by COMT [31], and binding with glutathione through GST occur [12, 28].

To improve candidate MDR inhibitor bioavailability and attenuate its metabolic disruption, several approaches have been implied such as injectable forms, other sophisticated drug delivery systems, combination with adjuvants like piperine and caffeine to diminish glucuronidation, and chemical modification of parent molecules to bypass efficient metabolic guardians [6, 24, 27].

A very high probability of drug-drug interactions between the adjuvant therapeutics for MDR inhibition and anticancer therapies themselves, as inducers of MDR, should also be taken into consideration [32]. It has been reported that potential MDR suppressors of herbal origin may easily interact with the same efflux (P-gp) and metabolic (*Cyp3A4*) pathways as anticancer agents do, resulting in opposite outcomes: inhibition or expression of MDR components, depending on timing, dosages, posology, and route of drug administration [33]. Recent findings have shown that this kind of drug-drug interaction is highly influenced by genetic polymorphisms of efflux proteins (MDR1) and metabolic enzymes (*Cyp3A5*) [34].

Emerging evidence shows that MDR could be an evolutionary defined mechanism to preserve normal and cancer stem cell populations. In this direction, redox signalling becomes a probable candidate to maintain cell stemness [1]. Therefore, the development of clinically efficient redox modulators of MDR should selectively target cancer stem cells, while leaving normal stem cells intact.

## 3. Redox Dependence of Acquired Multidrug Resistance: Modulation by Direct and Indirect Antioxidants

Most chemotherapeutic agents generate ROS, which bind to specific structures within the cancer cells and promote cell death. Chemotherapeutic agents disturb the redox homeostasis in cells and change their ability to cope with excessive ROS levels through the production of protective direct antioxidants [35]. Direct antioxidants are modified in this process and need to be resynthesised [36]. Glutathione (GSH) is considered as the main redox buffer in a cell because it supplies large amounts (millimolar concentrations) of reducing equivalents [37]. The intracellular thiol redox* status* is described as the ratio of reduced to oxidised forms of thiols (GSH/GSSG), which decreases under oxidative stress conditions, and GSH reversibly forms mixed disulfide bonds between protein thiols (S-glutathionylation) to prevent protein oxidation [38]. Besides GSH, thioredoxin (Trx), another important endogenous antioxidant, provides protection against oxidative stress [39, 40]. Nrf2 regulates Trx and sulfiredoxin enzymes, which are involved in the regeneration of the reduced form of nicotinamide adenine dinucleotide phosphate (NADPH) and synthesis of GSH [41]. Among the potential mediators of chemoresistance, Trx plays critical roles in the regulation of cellular redox homeostasis and redox-regulated chemoresistance [42–44].

In a recent study, Zhang et al. have shown that inhibiting Nrf2 expression through the transfection of shRNA plasmids in non-small-cell lung cancer cells significantly inhibited the expressions of glutathione pathway genes, antioxidants, and multidrug resistance proteins and induced the generation of ROS, decreased the level of GSH, and inhibited cell proliferation [45]. It has been reported that diffuse large B lymphoma cells expressed higher-than-normal basal levels of Trx, which was associated with decreased survival. Suppressed Trx inhibited cell growth and clonogenicity and sensitised the lymphoma cells to doxorubicin [44]. In a recent study, Raninga et al. [46] have reported the cytoprotective role of tTrx1 and thioredoxin reductase 1 (TrxR1) enzyme in multiple myeloma. Trx inhibitors were utilised in a variety of human cancers including acute myeloid leukemia [47], colorectal cancer [48], and lung cancer [49] to inhibit tumour growth and to stimulate ROS-induced apoptosis. Auranofin, a TrxR1 inhibitor, caused oxidative stress-induced cytotoxicity and apoptosis in cancers including chronic myeloid leukemia [50], chronic lymphocytic leukemia [51], prostate cancer [52], and breast cancer [53]. Signal transducer and activator of transcription 3 (STAT3) activation is commonly observed in multiple myeloma, chronic lymphocytic leukemia, gastric cancer, lung cancer, and laryngeal carcinoma. Dietary gamma-tocotrienol inhibited both induced and constitutive activation of STAT3 in multiple myeloma and prostate cancer cell lines [54]. High-dose intravenous ascorbate inhibited NADPH-oxidase and was selectively toxic to tumours with low CAT activity [55]. Recent study has reported that a naphthoquinone derivative induced cell death depending on Bax deficiency. In conclusion, it has been suggested that naphthoquinone might be clinically feasible to overcome chemoresistance [56].

Plant-origin polyphenols or their synthetic derivatives have been recognised as redox-active molecules with relatively low toxicity. Some of them, for example, luteolin, apigenin, and chrysin, exert both direct and indirect antioxidant effects by scavenging ROS and increasing Nrf2 activity, followed by the induction of its target antioxidant genes [57]. The natural polyphenols are also substrates for ABC transporters as they bind to the active sites of the transporters and reduce drug efflux [58]. The prooxidant capacity of some polyphenols (quercetin, epigallocatechin gallate) allowed their identification as chemotherapeutic adjuvants since they selectively enhanced cytotoxic effects of chemotherapeutics [59]. Shin et al. have reported that some specific polyphenols triggered cell cycle arrest and apoptotic cell death in cisplatin-resistant A2780/Cis human ovarian cancer cells [60].

### 3.1. Antioxidant-Associated Modification of Drug Transporting Systems

Elevated GSH levels trigger chemoresistance by different pathways: direct interaction with drugs and ROS, prevention of protein and DNA damage, and induction of DNA repair. For example, MRP1 causes efflux of some xenobiotics (e.g., vincristine, daunorubicin) through a cotransport mechanism with GSH [26, 61–63]. Oxidative stress was more cytotoxic towards B16 melanoma cells with low GSH concentrations [64]. Tumour cells overexpressing *γ*-glutamyl-transpeptidase were more resistant to H_2_O_2_ and chemotherapeutics, such as doxorubicin, cisplatin, and 5-fluorouracil [65]. GST-related chemoresistance modulated protein-protein interactions with members of the mitogen-activated protein (MAP) kinases including c-Jun N-terminal kinase 1 and apoptosis signal-regulating kinase 1 and altered balance of kinases during drug treatment [66]. This complex mechanism involved the interaction of promoter regions for* GST* and* GGT* with NF-*κ*B and Nrf2 followed by upregulation of several detoxification genes, such as ferritin, GSH-*S*-reductase, and hemeoxygenase-1. Hypoxia induced breast cancer resistance protein (BCRP) expression in tissues by interacting with heme and porphyrins thus increasing levels of cytoprotective protoporphyrins [67]. Overexpression of BCRP is known to induce resistance to various chemotherapeutic drugs, such as topotecan and methotrexate [68].

#### 3.1.1. MDR Induction and Possibility to Inhibit It by Redox-Active Substances Affecting Glutathione Metabolism

**Figure 3:**
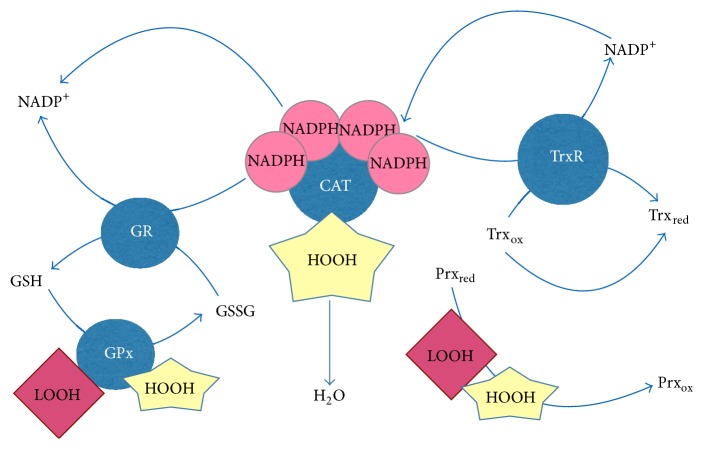
Antioxidant and prooxidant systems as molecular targets for redox-active MDR modulators. Glutathione and enzymes involved in the glutathione metabolism such as glutathione reductase (GR) and glutathione peroxidase (Gpx) as well as gamma-glutamyl cysteine ligase, catalase, NADPH-oxidase, thioredoxin (Trx), and peroxiredoxins (Prx) are potential molecular targets for future MDR modulators.

Definite redox-active compounds, such as quinones, polyphenols, oligomeric proanthocyanidins, ergothioneine, ovothiols, tannins, or terpenes, behave as redox modulators and trigger redox-related events, such as ROS increase and GSH depletion, causing apoptosis of cancer cells [69]. In general, redox modulations in cancer cells could initiate cell differentiation or could induce apoptosis [70]. Acetaminophen, a widely used drug to combat pregnancy-connected toxicity [71], induced ROS production in human choriocarcinoma cells by reducing BCRP and GSH content and activating Nrf2-targeted genes: NAD(P)H dehydrogenase, quinone 1 (NQO1), and hemeoxygenase-1. On the other hand, genetic knockout of* TrxR1* gene resulted in liver insensitivity to acetaminophen due to drastic disruption of the link between redox homeostasis and drug metabolism in the liver [72]. Glyoxalase 1, a key enzyme converting *α*-oxoaldehydes into corresponding *α*-hydroxy acids, has been found to be amplified in many primary tumours and cancer cell lines [73]. In this regard, Young et al. [73] have reported that overexpression of both enzymes glyoxalase 1 and transglutaminase 2, an enzyme catalysing polyamine conjugation/deamidation, led to increased tumour cell survival, drug resistance, and metastasis [74]. The use of photodynamic anticancer therapy is particularly attractive because of its specificity and selectivity [75]. Hypericin, a naphthodianthrone, is a promising photosensitizer, which is feasible for photodynamic therapy, for fluorescence diagnosis, and for topical applications [76]. Mikešová et al. [77] have shown that hypericin content in cells, GSH levels, and redox* status* correlated with hypericin-induced photocytotoxicity. In contrast, resveratrol attenuated cisplatin toxicity by maintaining GSH levels [78, 79]. It has also been demonstrated that buthionine sulfoximine, an inhibitor of GSH biosynthesis, increased the sensitivity of the cells to chemotherapeutics, while N-acetyl cysteine exhibited the reverse effect, particularly in drug-resistant cells [61, 62, 80]. Malabaricone-A, a diarylnonanoid with a potency of MDR reversal, induced depletion of GSH, inhibited GPx activity, and caused redox imbalance [81]. Collectively, molecular pathways-targets for MDR modulation by GSH controlling agents are schematically presented in Figure 3.

#### 3.1.2. Overexpression of ABC Transporters: Effects of NADPH-Oxidase and CYP Inhibitors

NADPH-oxidase (NOX) is an oxidoreductase and plays crucial roles in cell growth, proliferation, and regulation of phosphatases and transcription factors via redox-sensitive cysteine residues [82]. Elevated NOX expression has been shown in breast cancer [83], colon cancer [84], and neuroblastoma cells [85]. Barth et al. have demonstrated that pharmacological block of glucosylceramide synthase, a stimulator of NOX activity, substantially improved cytotoxicity of chemotherapeutics in glioblastoma cells [86]. The molecular mechanism of anticancer effects of cisplatin involves activation of Akt/mTOR pathway regulated by NOX-generated ROS, and NOX inhibition by diphenyl iodonium was critical for cisplatin cytotoxicity [87].

Overexpression of the drug and xenobiotic metabolising cytochrome P450 enzymes for a long time has been considered as one of the major mechanisms of chemoresistance in solid tumours [88, 89]. Types 1 and 2 CYPs have been proven to activate procarcinogens into ultimate carcinogens [90]. CYPs have become therapeutic targets in anticancer protocols amid their involvement in the activation and/or inactivation of chemotherapeutic drugs [91]. Molina-Ortiz et al. reported that altered CYP expression played a crucial role in the therapy of Rhabdomyosarcoma patients [92]. The* in vitro* antitumour action of natural product austocystin D has been explained by selective activation of CYP enzymes leading to DNA damage [93].

### 3.2. Chemotherapy-Induced Inflammatory Responses May Cause Redox-Regulated Multidrug Chemoresistance

To increase clinical efficacy of chemotherapy and combat MDR, molecular and cellular processes promoting inflammation have been targeted due to the common knowledge that (i) inflammatory cells are present within tumours and (ii) tumours arise at sites of chronic inflammation [94, 95]. Cancer promotion and progression stages are accelerated by ROS generated by immune cells, mediators of inflammation [96]. The induction of antioxidant defence enzymes in tumours as an adaptation to oxidative attacks from host immune cells might contribute to chemoresistance. Thus, hydrogen peroxide-resistant thymic lymphoma cells with increased catalase and total SOD activities, altered GSSG/GSH redox potential, and oxidised NADP^+^/NADPH pool exhibited resistance to conventional chemotherapeutics, such as cyclophosphamide, doxorubicin, vincristine, and glucocorticoids [97]. Chemotherapeutic agents caused release of ATP into the extracellular space as they induced tumour cell death [98]. Following accumulation of adenosine in tumours through CD39 and CD73, immune responses were suppressed [99]. Clayton et al. showed that exosome-expressing CD39 and CD73 suppressed T cells through adenosine production [100]. Oxidative stress has putative impact on the activation and regulation of protein kinase C (PKC) with redox-sensitive regions in both N-terminal regulatory domain and C-terminal catalytic domain. Rimessi et al. [101] have demonstrated that PKC*ζ* induced resistance to apoptotic agents following its translocation into the nucleus as a result of oxidative stress. Nuclear PKC*ζ* inhibitor restored the apoptotic susceptibility of doxorubicin-resistant cells by forming a complex with the proinflammatory transcription factor NF-*κ*B and promoting IL-6 synthesis, thus favouring tumorigenesis and MDR [102].

#### 3.2.1. Activation of NF-*κ*B-Dependent Pathways and Their Inhibition by Antioxidants

NF-*κ*B is the key transcription factor involved in the inflammatory pathway. NF-*κ*B is constitutively active in many of the signalling pathways implicated in cancer. Hyperactivation of NF-*κ*B in cancer cells promotes cancer cell survival by inducing the upregulation of antiapoptotic proteins such as MnSOD and Bcl-2 family members and the inhibition of proapoptotic proteins and is linked directly to the inflammation-induced chemoresistance. NF-*κ*B protects against oxidative stress and activates transcription factor c-myc,* MMP* gene expression, and tumour angiogenesis and remodels extracellular matrix, while NF-*κ*B inhibition blocks cell proliferation [95, 103–106]. NF-*κ*B is associated with aberrant growth, resistance to apoptosis, and overexpression of the genes involved in cell cycle promotion in cancer cells. In a recent study, it has been shown that isorhamnetin, a metabolite of quercetin, enhanced antitumour effects of chemotherapeutic drug capecitabine through negative regulation NF-*κ*B [107]. Singh et al. have reported that tea polyphenols inhibited cisplatin enhanced activity of NF-*κ*B [108]. FADD-like IL-1beta-converting enzyme inhibitory protein (FLIP) is a potent inhibitor of caspase-8-mediated apoptosis involved in NF-*κ*B activation. Talbott et al. revealed that FLIP regulates NF-*κ*B through protein S-nitrosylation, a key posttranslational mechanism controlling cell death and survival strategies [109]. Overexpression of cyclin D1 in cancer cells was reported in cisplatin chemoresistance. In contrast, reduction of cyclin D1 expression resulted in the increased sensitivity to cisplatin due to reduced NF-*κ*B activity and apoptosis [110]. It was shown that vitamin E compounds, such as *δ*- and *γ*-tocotrienol, inhibited NF-*κ*B activity, cell growth, cell survival, and tumour growth. In parallel, *δ*-tocotrienol augmented sensitivity of pancreatic cancer to gemcitabine [111].

Curcumin, a nontoxic food additive extensively used for food flavouring [112], has been found to suppress human hepatoma through inhibition of tumour cell proliferation, cell cycle arrest in G2/M phase, and induction of apoptosis [113]. Numerous publications have reported curcumin as a sensitiser for a number of anticancer drugs [114], first of all, cisplatin [115]. Results of recent studies have also suggested that curcumin was a reversal of induced MDR by multiple mechanisms such as the inhibition of ABC transporter expression and function, activation of ATPase, and modulation of NF-*κ*B activity during anticancer therapy [116]. In contrast, caffeic acid, a natural phenolic, prevented antiproliferative and proapoptotic effects induced by paclitaxel in lung cancer cells by the activation of NF-*κ*B-survivin-Bcl-2 axis, thus contributing to acquired MDR [117].

#### 3.2.2. Activation of Phosphoinositol-3 Pathway and Its Inhibition in a Redox Fashion

The phosphatidylinositol-3 kinase (PI3K) pathway has been widely considered to be associated with oncogenesis, cancer progression, and multiple hallmarks of malignancy [118]. Consistently, PI3K pathway is a common mechanism of resistance to antineoplastic agents [119]. Of note, resistance to PI3K inhibitors may also develop due to aberrant compensatory signalling through other pathways [120]. The three main molecules in this pathway are PI3K, Akt, and mammalian target of rapamycin (mTOR). Recently, it has been reported that PI3K-mTOR inhibitor enhanced the cytotoxicity of temozolomide, an advanced chemotherapy for malignant gliomas [121].

Since activation of integrins, proteins expressed on the cytoplasmic membrane of malignant cells, is controlled directly by a redox site by disulfide exchange in their extracellular domain, redox modifications of thiols could alter essential functions of integrins [122]. It was suggested that inactivation of VLA-4 integrin by nontoxic tellurium compound was due to its binding to the thiol groups of cysteines that decreased PI3K/Akt/Bcl-2 signalling while enhancing drug sensitivity [123]. Gao et al. demonstrated that the natural bioflavonoid apigenin reversed drug-resistant phenotype by its suppressor effect on PI3K/Akt/Nrf2 pathway in doxorubicin-resistant Nrf2 overexpressing cells [124].

#### 3.2.3. Activation of Toll-Like Receptors (TLRs) by Chemotherapeutics and Inhibitory Effects of Redox Modulators

Recent studies implicate bacterial, parasitic, and viral infections as a possible link between inflammation and carcinogenesis [125]. One possible redox-sensitive signalling pathway connecting infection-associated inflammation and carcinogenesis is mediated by Toll-like receptors (TLRs). The hypothesis is that bacterial products, such as lipopolysaccharide, could activate TLR4-MyD88 axis in tumour cells followed by the production of proinflammatory cytokines, overexpression of antiapoptotic signals (XIAP and pAkt), and, finally, acquisition of chemoresistance by ovarian cancer cells [126]. Both* in vivo* and* in vitro* experiments have shown that anticancer drug paclitaxel exerted two opposite modes of action: killing of breast cancer cells and enhancement of their survival through activation of TLR4 pathway [127]. The authors suggested that simultaneous TLR4 block could reverse MDR to paclitaxel and improve efficacy of the anticancer therapy.

Activation of TLRs in cancer cells seems to contribute to the tumour growth, cancer cell survival, and MDR via a signalling cascade involving cytokine/chemokine production [128]. It was shown that ligation to the TLR2 in lung cancer cells induced activation of mitogen-activated protein kinases (MAPK) and NF-*κ*B, a classical pathway of survival strategy [129]. Stimulation of TLR7/TLR8 in pancreas cancer cells resulted in elevated NF-*κ*B and COX-2 expression, increased cancer cell proliferation, and reduced chemosensitivity [130]. Active TLR-4/MyD88 signalling was also found in epithelial ovarian cancer cells and influenced the drug response [131]. The relationships between the expressions of TLR-4, MyD88, and NF-*κ*B have been examined in epithelial ovarian cancer patients. Increased MyD88 expression was found to be associated with poor survival rate [132].

### 3.3. Chemotherapy-Induced Redox-Dependent Stress Responses Leading to Adaptation

Along with killing cancer cells, chemotherapeutic agents induce their stress and adaptive responses. Signalling pathways and gene expression in response to chemotherapeutics play pivotal roles in the development of acquired MDR [133]. Key functional aspects of cellular stress response include damage to membrane lipids, proteins, and DNA and alterations in the redox* status*, energy metabolism, cell cycle, and proliferation [134]. Thus, there is clear-cut evidence that upregulation of nonenzymatic and enzymatic antioxidant defence, molecular chaperones, and stress responsive proteins are responsible for acquired MDR [135]. These molecular pathways are potential targets to enhance the cytotoxic effects of chemotherapeutics and to overcome drug resistance.

#### 3.3.1. Nrf2 as a Perspective Target to Overcome MDR in Tumours

Nrf2, a redox-sensitive transcription factor, plays a crucial role in redox homeostasis during oxidative stress. Nrf2 is sequestered in cytosol by an inhibitory protein Keap1 causing its proteosomal degradation [136]. In response to oxidative stress, Nrf2 translocates to nucleus and binds to ARE that increases the expression of antioxidant genes such as hemeoxygenase-1, NAD(P)H: quinone oxidoreductase 1, aldo-keto reductases, and several ATP-dependent drug efflux pumps [137]. Many genes involved in phase II metabolism are also induced by Nrf2, including GSTs, UGT, and UDP-glucuronic acid synthesis enzymes [138]. While Nrf2 upregulation causes chemoresistance, its blockade sensitises a variety of cancer cells, including neuroblastoma, breast, ovarian, prostate, lung, and pancreatic cancer cells, to chemotherapeutic drugs [139]. Several flavonoid compounds have been reported to be potent Nrf2 inhibitors, such as epigallocatechin 3-gallate, luteolin, and brusatol [140, 141]. Nrf2 was upregulated in hepatocellular carcinoma and positive correlation was found between Nrf2 expression and antiapoptotic Bcl-xL and MMP-9 [142]. Quercetin treatment increased the total cellular amount and nuclear accumulation of Nrf2 protein in malignant mesothelioma cells [143].* In vitro* suppression of Keap1 in human prostate and non-small-cell lung carcinoma cell lines elevated Nrf2 activity and increased sensitisation to various chemotherapeutic agents and radiotherapy [144, 145]. These results demonstrated that Nrf2 inhibitors are effective adjuvants of chemotherapeutic drugs.

### 3.4. Chemotherapy-Induced Prosurvival and Antiapoptotic Cellular Strategies: Roles for Anti- and Prooxidants

Cellular redox homeostasis is maintained by the balance between endogenous antioxidant defence system, including antioxidant enzymes such as SOD, catalase (CAT), GPX, GSH, proteins, and low-molecular-weight scavengers, such as uric acid, coenzyme Q, and lipoic acid, and the prooxidant molecules, leading to the formation of several highly oxidising derivatives.

#### 3.4.1. p53 Proapoptotic Protein

p53 is considered as the guardian of the genome, and several gene mutations encoding p53 have been detected in several tumour cells. Under physiological conditions, activated p53 plays a key role in tumour prevention by promoting synthesis of antioxidant enzymes. ROS-induced DNA damage activates p53, leading to apoptosis via the mitochondrial intrinsic pathway and increasing the synthesis of prooxidant enzymes. Since p53 is a redox-sensitive factor, ROS negatively modulates its activity via oxidative modification of the cysteine residues at the DNA-binding site. It has been proposed that the loss of p53 function in cancer cells is associated with their ability to avoid apoptosis [146]. Mutations of p53 are involved in resistance to chemotherapy [147]. It was demonstrated that p53 regulated Nrf2 negatively and interfered with the ability of Nrf2 to bind to DNA. A low level of p53 favoured binding of Nrf2 to DNA. In a recent study, the treatment with bortezomib, which is a selective proteasome inhibitor, induced Myelocytomatosis Viral Oncogene Neuroblastoma (MYCN) downregulated p53 expression, leading to cell survival in neuroblastoma [148].

#### 3.4.2. Bcl-2 Antiapoptotic Protein

Bcl-2 protein is a member of a family of apoptosis-modulating proteins which protects against a variety of apoptotic stimuli, mainly acting at the mitochondrial level. In addition to its antiapoptotic action, Bcl-2 has been shown to exert potent antioxidant effects [149] such as protection of lipid membranes against peroxidation reactions, maintenance of cellular redox status (i.e., NADH/NAD^+^ and GSH/GSSG in a reduced state) in response to oxidative stress [150], and elevation of cellular levels of glutathione and reducing equivalents [151]. Bcl-2 upregulates antioxidant defence systems, and its mitochondrial localisation contributes to achieving this effect. Bcl-2 is capable of forming ion channels as a regulator of mitochondrial permeability transition [152]. Mitochondrial permeability transition blockade prevents release of cytochrome c, an apoptosis initiating factor. Herrmann et al. reported that Bcl-2 selectively regulates nuclear localisation of cell death regulators such as p53 and NF-*κ*B [153]. Cellular redox state via thiols plays a major role in regulating mitochondria-mediated events during apoptosis, and oxidation of mitochondrial thiols is an apoptotic sensor. GSH is involved in detoxifying reactions and it has a high intracellular concentration. It serves as the major reducing peptide within all of the cells, due to its sulfhydryl group buffering and removing free radicals generated during metabolic processes such as respiration. GSH is an important factor for Bcl-2 ability to suppress apoptosis. Chaiswing et al. observed that thiol redox* status* and activities and expression of several antioxidant enzymes exhibited distinct patterns in two prostate cancer cell lines at different growth phases, suggesting that modulation of thiol redox status might be useful as a therapeutic tool to modify cancer cell proliferation and tumour aggressiveness [154]. Mitochondrial metabolism is altered in malignant cells so that, in contrast to normal or benign cells, malignant cells accumulate citrate due to low activity of mitochondrial aconitase, become citrate-oxidising cells, and exhibit low amounts of citrate. The higher rate of mitochondrial substrate metabolism explains the increased levels of ROS associated with malignancy and metastasis. The foregoing discussion illustrates the importance of GSH in mitochondrial function and redox* status*, in determining the metastatic aggressiveness and sensitivity of cancer cells to chemotherapeutic agents, and provides the rationale for mitochondrial GSH as a potential therapeutic target in cancer [155].

### 3.5. Chemotherapy-Induced Transcription and Activity of Aromatic Hydrocarbons (Ah) Receptor

AhR is a basic helix-loop-helix transcription factor that, prior to ligand binding, is stabilised in the cytoplasm by direct interaction with several proteins such as heat shock protein 90, its cochaperon, and X-associated protein 2. Upon binding to aromatic ligands, toxins, drugs, phytochemicals, and sterols, the AhR-ligand complex shuttles from cytoplasm to the nucleus, where it heteromerises with the AhR nuclear translocator (Arnt) to form the transcription complex able to bind to xenobiotic responsive elements (XRE) DNA-binding motifs located in the promoter region of the target drug metabolising genes, such as phase I (mainly,* Cyp1* subfamily) and II metabolising enzymes and Nrf2 [156–158]. One of the downstream targets for AhR is BCRP encoded by* ABCC3* gene [159–161]. The coordinate AhR- and Nrf2-dependent transcriptional regulation of human UGTs by utilising both XRE- and ARE-binding motifs takes place to protect cells from xenobiotic and oxidative stresses [162]. The elegant study with genetically modified mice has clearly demonstrated that Nrf2 is required for ligand-associated induction of classical “AhR battery” genes NQO1, GST isoforms, and UGTs [163]. Apart from metabolic enzymes, a number of growth factors, cytokines, chemokines, and their receptors are downstream gene targets for activated AhR [164, 165]. AhR is also functionally connected with epidermal growth factor receptor, presumably, through NF-*κ*B-regulated pathway [166], thus influencing the epithelial cell proliferation. AhR can also cross-talk and directly interact with proteins involved in major redox-regulated signalling pathways such as NF-*κ*B and various kinases such as Src, JNK, p38, and MAPK [167] and with oestrogen receptors to mediate oestrogen metabolism [168, 169]. Recent studies have unraveled unsuspected physiological roles and novel alternative ligand-specific pathways for this receptor that allowed hypothesising numerous pharmacological roles of AhR ligands useful for the development of a new generation of anti-inflammatory and anticancer drugs [159, 170].

The AhR-mediated regulation of aromatic hydrocarbons metabolism has been implicated in a variety of cancers [164, 171] affecting different stages of carcinogenesis. If metabolic activation of the organic molecules increased the levels of their adducts with DNA thus promoting cancer initiation, anticancer drug- or toxin-induced AhR activation played a pivotal role in cancer promotion and progression [172, 173]. Elevated AhR expression associated with constitutive nonligand activation has been found in several cancers as evidenced by the nuclear localisation of AhR and induced downstream gene* Cyp1A1* [174, 175]. Stable knockdown of AhR decreased the tumorigenic and metastatic properties of breast cancer cell line* in vitro* and* in vivo*. On the other hand, AhR overexpression in nontumour human mammary epithelial cells transformed them in cells with malignant phenotype [176]. Of importance, AhR knockdown downregulated the expression of* ABCC3*; overexpression of this gene in breast cancer has been strongly associated with acquired MDR [177] and resistance to paclitaxel, a drug widely used in the treatment of metastatic breast cancer [178]. Inherited polymorphisms in AhR, for example, substitution Arg554Lys, and its machinery [179, 180] or presence of endogenous ligands-stimulators for the receptor (cAMP, bilirubin, prostaglandins, oxidative lipids, etc.) could be implicated into inherited MDR.

To suppress AhR transcription pharmacologically several approaches have been proposed, including the modulation of protein-protein interaction between transcription factor, coactivators, and corepressors [160, 167, 181]. A number of dietary polyphenols with redox properties (resveratrol, quercetin, curcumin, etc.), indoles, tryptophane metabolites, bilirubin, and oxidised products of lipid metabolism have been suggested as nontoxic ligands-activators or ligands-inhibitors of AhR expression by competitive and noncompetitive pathways [181, 182]. If ligands-activators of AhR are regarded as potential anti-inflammatory agents [181–184], redox-active ligands able to suppress AhR expression/functions could be candidates for MDR reversals. For example, 7-ketocholesterol, a major dietary oxysterol, may actually strongly inhibit AhR activation [185]. Alpha-naphthoflavone is considered as a classical AhR antagonist blocking activation of XRE-containing reporter gene and* Cyp1* upregulation in hepatoma cells [186]. However, alpha-naphthoflavone is also a partial agonist of AhR and acts as a competitive inhibitor exclusively in the presence of another agonist. Recently, more selective pure ligands-antagonists of AhR have been developed such as 2-methyl-2H-pyrazole-3-carboxylic acid (2-methyl-4-o-tolylazo-phenyl)-amide, 3′-methoxy-4′-nitroflavone, 3′,4′-dimethoxyflavone, and 6,2′,4′-trimethoxyflavone. These were able to block the induction of* Cyp1A1*-dependent ethoxyresorufin* O*-deethylase (EROD) activity [187–190]. They all belong to redox-active flavones and after proper clinical studies on safety and efficacy could be feasible for combinatory anticancer therapy to combat MDR. Among a number of plant polyphenols used for topical application, exclusively the phenylpropanoid verbascoside and flavonoid quercetin proved to be strong inhibitors of UV- or FICZ-upregulated AhR-*Cyp1A1-Cyp1B1* axis in human keratinocytes [184], suggesting their potency as topical MDR suppressors/reversals.

## 4. Conclusions

The multiple pleiotropic interactions of redox-active molecules so far demonstrated on the molecular pathways controlling cellular MDR, from xenobiotic cellular uptake inhibition to the modulation of phase I/II enzyme detoxification, to the inhibition of Toll-like receptor activation and/or AhR expression and function, provide a consistent rationale for the necessity of more intense and systematic research efforts in the field of anti-/prooxidant adjuvants for anticancer chemotherapy, to attempt clinically effective MDR inhibition with tolerable toxicity.

The design and implementation of more selected and targeted clinical studies centred on redox-active candidate MDR-interfering molecules will possibly contribute to overcoming the presently dominating clinical practice, which confers a constantly growing interest in redox modulators and antioxidants as a mere palliative against the potent cytotoxicity of conventional and biologic anticancer drugs.

## Abbreviations

ABC: ATP-binding cassette
AhR: Aryl hydrocarbon receptor
Akt: Protein kinase B
AP-1: Activator protein 1
ARE: Antioxidant response element
*Bcl2*: Apoptosis regulator Bcl-2
BCRP: Breast cancer resistance protein
CAT: Catalase
COMT: Catechol-O-methyl transferase
CYP: Cytochrome P450
EM: Extensive Metabolisers
FLIP: FADD-like IL-1beta-converting enzyme inhibitory protein
GPx: Glutathione peroxidase
GSH/GSSG: Reduced glutathione/oxidised glutathione
GST: Glutathione-S-transferase
JNK1: c-Jun N-terminal kinase 1
Keap 1: Kelch-like ECH-associated protein 1
MDR: Multiple drug resistance
MMP: Matrix metalloproteinase
mTOR: Mammalian target of rapamycin
MXR: Multiple xenobiotic resistance
NADPH: Nicotinamide dinucleotide phosphate, reduced form
NAT: N-Acetyl transferase
NF-*κ*B: Nuclear factor kappa B
NQO1: NAD(P)H: quinone oxidoreductase 1
NOX: NADPH-oxidase
Nrf1/2: Nuclear factor E2-related factor 1/2
P-gp: P-glycoprotein
PPI3K: Phosphoinositol-3-kinase
PKC: Protein kinase C
PM: Poor Metabolisers
Prx: Peroxiredoxins
RNS: Reactive nitrogen species
ROS: Reactive oxygen species
SNP: Single nuclear polymorphism
SOD: Superoxide dismutase
STAT3: Signal transducer and activator of transcription 3
SULT1A1/A3: Sulfotransferases A1/A3
TNF*α*: Tumour necrosis factor alpha
TLR: Toll-like receptor
Trx: Thioredoxin
TrxR: Thioredoxin reductase
UGT: UDP-glucuronosyltransferase
UM: Ultrarapid Metabolisers
XRE: Xenobiotic responsive elements.
